# Effects of In Vitro Gastrointestinal Digestion on the Antioxidant Capacity and Anthocyanin Content of Cornelian Cherry Fruit Extract

**DOI:** 10.3390/antiox8050114

**Published:** 2019-04-30

**Authors:** Luminita David, Virgil Danciu, Bianca Moldovan, Adriana Filip

**Affiliations:** 1 Research Center for Advanced Chemical Analysis, Instrumentation and Chemometrics (ANALYTICA), Faculty of Chemistry and Chemical Engineering, Babeş-Bolyai University, 11 Arany Janos Street, 400028 Cluj-Napoca, Romania; muntean@chem.ubbcluj.ro (L.D.); danciuv@chem.ubbcluj.ro (V.D.); 2 Department of Physiology, Iuliu Hatieganu University of Medicine and Pharmacy, 1-3 Clinicilor Street, 400006 Cluj-Napoca, Romania; gabriela.filip@umfcluj.ro

**Keywords:** cornelian cherry, antioxidant capacity, anthocyanins, gastrointestinal digestion

## Abstract

Red fruits are considered a major source of antioxidant compounds in the human diet. They usually contain anthocyanins, phenolic pigments that confer them multiple health-promoting properties. The health benefits of these bioactive phytocompounds are strongly related to their bioavailability, which has been reported to be low. The aim of the present study is to investigate the changes in antioxidant capacity and anthocyanin content of Cornelian cherry fruit extract during gastrointestinal digestion. Thus, the work was designed using a simulated in vitro digestion model. The antioxidant capacity (AA) was tested by the 2,2-azinobis (3-ethylbenzothiazolyne-6-sulphonic acid) radical cation (ABTS) method, while quantification of anthocyanins (TAC) was accomplished by the means of the pH differential method and high performance liquid chromatography (HPLC). The results showed that gastric digestion had no significant effect on the TAC of the extract, while the AA slightly increased. After duodenal digestion, only 28.33% of TAC and 56.74% of AA were maintained. Cornelian cherries’ anthocyanins were stable in stomach, so they can be absorbed in order to manifest their antioxidant capacity at the cellular level. The duodenal digestion dramatically decreased the TAC and AA level in the fruit extract.

## 1. Introduction

**Figure 1:**
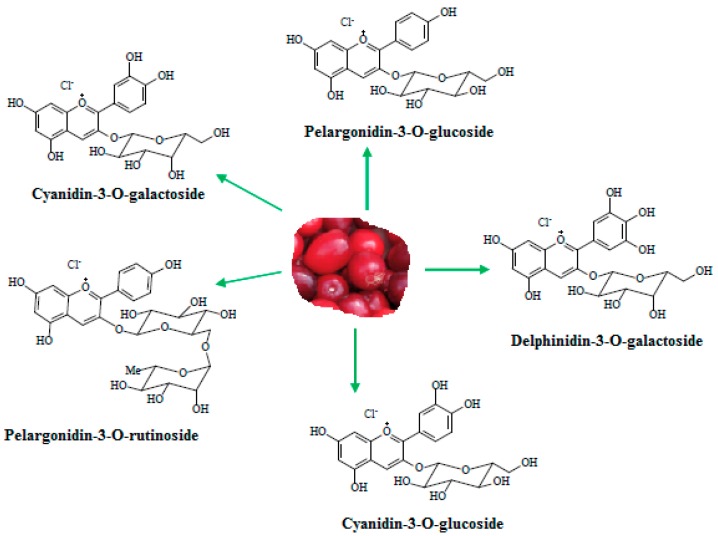
Structure of anthocyanins from Cornelian cherry fruit.

Cornelian cherry (*Cornus mas* L.) is a plant belonging to the genus *Cornus*, growing in southeastern Europe and Asia. Its fruits possess a sour tart taste, contain a single stone, and are usually eaten raw or processed as jams, liquors, vinegars, compotes, or marmalades [1,2,3]. These fruits are reported to contain biologically-active compounds, such as phenolic acids, flavonoids, iridoids, triterpenoids, organic acids, and vitamin C [4,5,6]. The ripe fruits of *Cornus mas* have usually an attractive red color, which is due to the presence of anthocyanins, plant secondary metabolites responsible for the red, purple, or blue color of fruits, vegetables, or flowers. Anthocyanins have recently drawn much attention due to their numerous health-promoting properties, such as antioxidant, anti-inflammatory, antidiabetic, antiobesity, neuroprotective, and antiatherosclerotic effects [7]. In Cornelian cherry fruits, several anthocyanins, such as pelargonidin and cyanidin derivatives, were identified (Figure 1). Du and Francis reported the presence of five anthocyanins: cyanidin-3-*O*-galactoside, cyanidin-3-*O*-rhamnosylgalactoside, delphinidin-3-*O*-galactoside, pelargonidin-3-*O*-galactoside, and pelargonidin-3-*O*-rhamnosylgalactoside [8,9]. Later studies indicated that Cornelian cherry fruits contain normally a mixture of three to five anthocyanic pigments identified by different techniques such as high performance liquid chromatography, mass spectrometry, or nuclear magnetic resonance. The anthocyanin composition is strongly related to Cornelian cherry cultivar. Kucharska et al. confirmed the presence of five anthocyanins, delphinidin, pelargonidin, and cyanidin-3-*O*-glycosides, by investigating 26 Cornelian cherry cultivars from Poland and Ukraine [10], while Moldovan et al. reported the presence of cyanidin-3-*O*-galactoside, pelargonidin-3-*O*-glucoside, and pelargonidin-3-*O*-rutinoside in a Romanian Cornelian cherry cultivar [11]. 

The dietary consumption of anthocyanin-rich fruits or their derivative products may prevent numerous diseases and improve human health. Mazza et al. reported an increase in the antioxidant status of the human serum after oral intake of blueberries anthocyanins [12], although earlier studies reported that only 1% of anthocyanin compounds are present in human plasma after fruit consumption [13]. After oral administration, anthocyanins can be absorbed in stomach, as well as in intestines, either in their intact form or as metabolites [14]. After absorption, anthocyanins are transported to heart, brain, liver, kidneys, or other tissues where they can exert their antioxidant capacity and their health-promoting properties. All these results indicate that investigating anthocyanin stability in the gastrointestinal tract could deliver important information on their bioavailability and their health beneficial properties. Simulated human gastrointestinal digestion studies demonstrated that anthocyanins are rather stable in stomach, but easily degradable in small intestine mainly due to the high value of the pH [7,15,16]. 

The main objective of this study was to evaluate the impact of the gastrointestinal tract’s biochemical conditions on the antioxidant compounds responsible for the beneficial effects on human health of Cornelian cherry fruits. Therefore, a simulated in vitro human digestion model was applied, and the stability of anthocyanins from the investigated fruits, as well as the changes in the antioxidant activity of the digested fruit extract were investigated. Although the anthocyanin profile, the total phenolic content, and the in vitro antioxidant capacity of the Cornelian cherry fruits were already elucidated by several research groups [10,11,17,18,19,20,21], this is the first study dealing with the impact of gastrointestinal digestion on their antioxidant activity and on the bioaccessibility of their anthocyanins. 

## 2. Materials and Methods

### 2.1. Chemicals

Cyanidin-3-*O*-galactoside, pelargonidin-3-*O*-glucoside, and pelargonidin-3-*O*-rutinoside standards were purchased from Extrasynthese (Lyon, France), and pancreatin from porcine pancreas and pepsin from porcine stomach were purchased from Alfa Aesar (Karsluhe, Germany). All other chemicals and solvents were obtained from Merck (Darmstadt, Germany) and were of analytical or HPLC purity. A TYPDP1500 water distiller (Techosklo LTD., Drzkov, Czech Republic) was used to obtain the distilled water.

### 2.2. Preparation of Fruit Extract

Cornelian cherry (*Cornus mas* L.) fruits were manually collected at ripening stage in August 2018 in Chinteni, Cluj County, Romania. The picked fruits were selected according color, mass, and shape uniformity and stored at −18 °C until further investigation. The Cornelian cherries’ anthocyanins were isolated by extraction applying an already published procedure [11]. Briefly, 90 g of homogenized fruit pulp were treated with 300 mL of food-grade acetone. The extraction was carried out at room temperature, under vigorous stirring for 60 min. The mixture was vacuum filtered through Whatman No. 1 filter paper, and the obtained solution was concentrated to a 65-mL final volume at 40 °C by a rotary evaporator to remove the acetone. The obtained concentrate was further subjected to determination of total anthocyanin content by the pH differential method, to identify anthocyanins present in the extract by HPLC analysis and in in vitro simulated gastrointestinal digestion.

### 2.3. Quantification and Identification of Anthocyanins

The total anthocyanin content of the Cornelian cherries’ anthocyanin-rich extract was determined using the well-known spectrophotometric pH differential method [22] as previously described [23]. Briefly, samples of fruit extract were mixed with 0.025 M potassium chloride buffer solution (pH = 1) and 0.04 M sodium acetate buffer solution (pH = 4.5), respectively. The absorbance of each resulting solution was measured at 506 nm and 700 nm, respectively, against distilled water as a blank, using a UV-Vis Perkin Elmer Lambda 25 double-beam spectrophotometer (Perkin Elmer, Shelton, CT, USA). The total anthocyanin content was expressed as cyanidin-3-glucoside equivalents/liter. All measurements were performed at room temperature, in triplicate. 

The identification of anthocyanins in the Cornelian cherry fruit extract was accomplished by HPLC analysis. For this purpose, an Agilent 1200 (Agilent Technologies Inc.; Santa Clara, CA, USA) equipped with a diode array detector HPLC system was used. The separation of anthocyanins from the fruit extract was performed on an Eclipse XTB-C18 (Agilent) column (150 × 4.6 mm inner diameter, particle size 5 μm), maintained at 20 °C. Samples of the extract were 2-fold diluted with 0.1% formic acid, filtered through a 0.2-μm PTFE membrane disk filter, and 20 μL were injected into the column. A mixture of two solvents, 0.1% formic acid aqueous solution (Solvent A) and 0.1% formic acid acetonitrile solution (Solvent B), was used as a mobile phase at a flow rate of 0.4 mL/min. The elution was performed by applying the following gradient profile: 0–16 min 95% A, 16–17 min 60% A, 17–20 min 5% A, and 20–21 min 95% A. The detection was performed at 506 nm. The main anthocyanins from the Cornelian cherry fruit extract were identified according to the consistency of the retention times compared to authentic anthocyanin standards. The quantification of each anthocyanin present in the extract was performed using a calibration curve of cyanidin-3-*O*-galactoside by an external standard method. All samples were injected in triplicate.

### 2.4. Evaluation of the Antioxidant Activity Using the ABTS Assay

The antioxidant capacity of the Cornelian cherry fruit extract was determined by the ABTS radical scavenging activity method [24] with some modifications [25]. The ABTS•^+^ stock solution was freshly diluted with distilled water to prepare a working solution with an absorbance between 0.6 and 0.8 recorded at 734 nm against distilled water using a spectrophotometer. The free radical scavenging capacity of the fruit extract was evaluated by adding 0.1 mL of extract sample to 6 mL diluted ABTS solution followed by incubation at room temperature, in the dark, for 15 min. After 15 min, the absorbance of the sample was monitored at 734 nm, and the antioxidant activity was calculated using a calibration curve of Trolox standard and expressed in μmol Trolox equivalents/L.

### 2.5. In Vitro Simulated Digestion Process

The gastrointestinal digestion was simulated according to a slightly modified protocol of Gil-Izquierdo [26]. Samples of 50 mL were subjected to an in vitro gastric digestion process by adjusting the pH of the extract to 2, by adding 1 M HCl solution. Thereafter, pepsin from porcine gastric mucosa (15.750 units EC 3.4.23.1) was added, and the samples were incubated in the absence of light, in a shaker (200 rpm), at 37 °C, for 2 h. After the simulated gastric digestion, aliquots of 5 mL were collected, rapidly cooled at −20 °C, and used further for the evaluation of anthocyanin composition and antioxidant activity of the gastric digested samples. The remaining solution from the gastric digestion step was further submitted to the in vitro simulated intestinal digestion process. The pH of the remaining solution was adjusted to 7.5 with 1 M NaHCO_3_ solution. Then, 22.5 mL of pancreatin (2 mg/mL) from porcine pancreas (8 × USP specifications) and bile salts (25 mg/mL) solution were added, and the obtained mixture was further incubated at 37 °C for another 2 h. The obtained digested samples were properly diluted with 0.1% formic acid (3-fold for the gastric digestion samples and 1.6-fold for the intestinal digestion samples) and analyzed directly by HPLC for the determination of the anthocyanin profile, and also, the antioxidant capacity and the total anthocyanin content were evaluated. The simulation of the in vitro digestion process was performed in triplicate. 

### 2.6. Statistical Analysis

All reported data are presented as the mean values ± the standard deviation obtained from the three replicates. Statistical analysis was conducted using XLSTAT Release 10 (Addinsoft, Paris, France) software, and the level of significance was evaluated by one-way analysis of variance (ANOVA), considering *p*-values < 0.05 to be significant. 

## 3. Results and Discussion 

**Figure 2:**
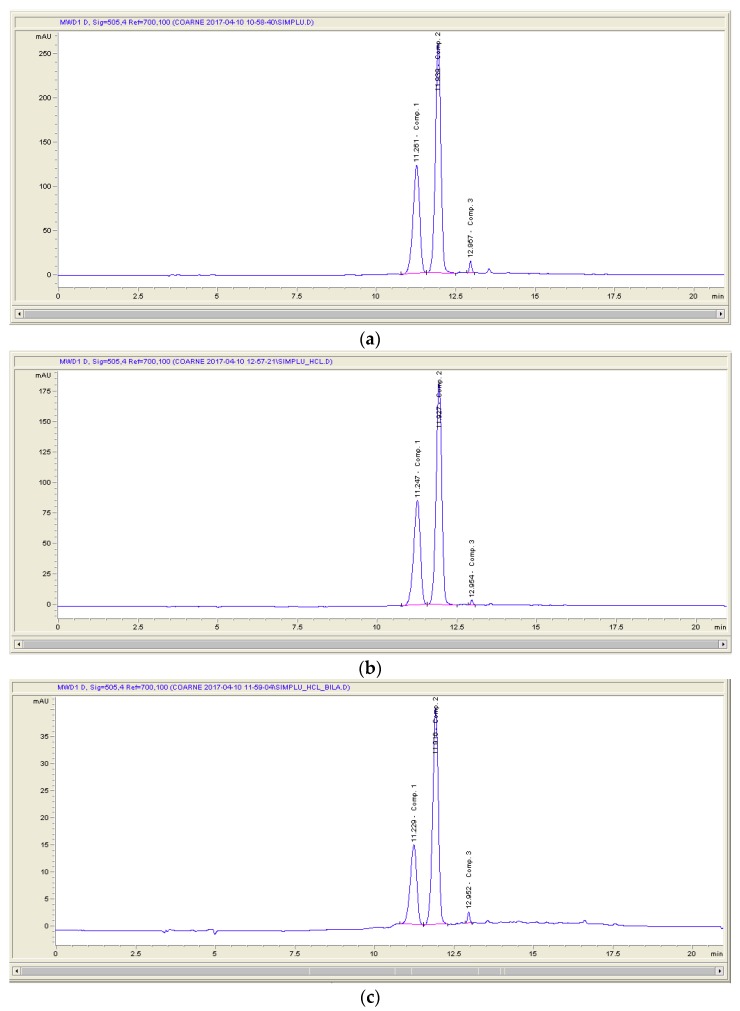
HPLC chromatograms (520 nm) of Cornelian cherries’ anthocyanins before and after in vitro digestion: (**a**) crude extract; (**b**) gastric digestion; (**c**) intestinal digestion.

Cornelian cherry fruits have been reported as a valuable source of anthocyanins, compounds with high antioxidant activities that contribute to the beneficial biological properties of these fruits. A diet rich in anthocyanins results in downregulating disease markers, improves the antioxidant function, and protects cells against the deleterious effects of oxidative stress [27]. The antioxidant activity of the Cornelian cherries’ extract was evaluated using the ABTS assay and was found to be 553 μM Trolox. The anthocyanin content was determined by the pH differential method and was 360 mg Cy-3-glu equivalents/L. The HPLC analysis of the Cornelian cherry fruit extract revealed the presence of three anthocyanins (Figure 2). The results were in agreement with those previously reported by Moldovan et al. [11]. The three main anthocyanin peaks detected at 506 nm were identified by comparing the retention times and the UV absorption spectra with authentic anthocyanin standards and were assigned to cyanidin-3-*O*-galactoside (Peak 1), pelargonidin-3-*O*-glucoside (Peak 2), and pelargonidin-3-*O*-rutinoside (Peak 3).

The content of the three anthocyanins is presented in Table 1. The main anthocyanin of the Cornelian cherry fruits was pelargonidin-3-*O*-glucoside (62.99% from total anthocyanin content), followed by cyanidin-3-*O*-galactoside (35.68% from total anthocyanin content). Pelargonidin-3-*O*-rutinoside has been identified in very small amounts (<2%) in the crude fruit extract.

**Figure 3:**
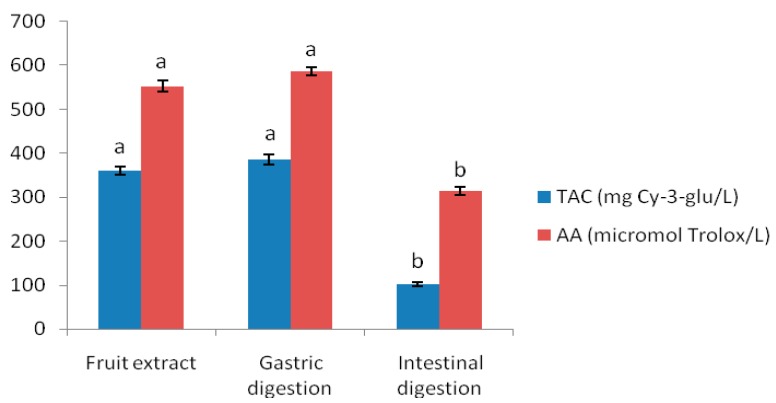
Changes in total anthocyanin content and antioxidant activity of Cornelian cherry fruit extract before and after in vitro gastrointestinal digestion. Means (bar value) with different letters are significantly different (*p* < 0.05).

Anthocyanins are generally not very stable compounds. Many factors can affect the stability of these antioxidant compounds, such as pH, oxygen, chemical structure, and the presence of enzymes [28]. Thus, investigating their stability during their passage through the gastrointestinal tract is very important, in order to understand the bioavailability of these compounds. In the present study, the stability of Cornelian cherry anthocyanins was investigated using an in vitro simulated gastrointestinal digestion model. The Cornelian cherry fruit extract was incubated with pepsin at pH = 2 for the gastric digestion and, after that, with pancreatin and bile salts at pH = 7.5, to simulate small intestine digestion. After each step of the in vitro digestion, samples were analyzed in terms of their total anthocyanin content and antioxidant capacity. HPLC analysis was conducted to monitor changes in anthocyanin composition and content after each digestion step. Stomach digestion did not significantly change the qualitative or quantitative composition of the anthocyanin compounds. Figure 2b presents the HPLC chromatogram after simulated gastric digestion, and no differences between crude extract and that subjected to digestion in gastric conditions were observed. After subjecting the anthocyanin-rich extract to simulated gastric digestion, a slight increase of the total anthocyanin content to 386 mg/L was noticed. The same anthocyanins as in the original sample were recovered after stomach digestion, the recovery being 107.23%. Our findings are in good agreement with those obtained in other studies. Ryu et al. reported a slight increase after gastric digestion of Bokbunja anthocyanins [15], while Sun et al. observed the same result in the case of purple rice anthocyanins subjected to in vitro simulated digestion [29]. These findings strongly suggest that Cornelian cherry anthocyanins are highly stable when exposed to stomach conditions. The low pH value in stomach mainly contributes to the high stability of anthocyanins, which at this pH (pH = 1.5–2), occurs in the chemical structure of a stable flavylium cation [30]. The increase of the anthocyanin content could also suggest that an acidic environment and digestive enzymes improve the release of the monomeric anthocyanins from the polymeric ones, by disrupting macromolecules. Additionally, an increase in the antioxidant activity during simulated gastric digestion occurred (Figure 3). Changes in total anthocyanin content and radical scavenging capacity were not significant during the in vitro gastric digestion as compared to undigested extract (*p* > 0.05).

The samples obtained after gastric digestion were further transferred to a mild alkaline intestinal environment, and pancreatic enzymes and bile salts were added in order to simulate the intestinal digestion process. After small intestine digestion, a clear decrease of the total anthocyanin content up to 26.46 mg cyanidin-3-*O*-glucoside equivalents/L was observed, reduced by more than 70% compared to the undigested sample. The low recovery of anthocyanins after intestinal digestion (26.46%) can be explained by the low stability of these compounds under alkaline conditions (pH = 7.5) attributed to the structural changes of the flavylium cation to a colorless, less stable chalcone [30]. Other studies also found that simulated intestinal digestion had a dramatic impact on the anthocyanins from different fruits. Pomegranate juice anthocyanins decreased by ~97% during this stage of digestion [31], while blueberry anthocyanins decreased more than 80% after intestinal digestion [32]. All the anthocyanin compounds present in the Cornelian fruit extract presented a significant decrease during incubation under small intestine conditions. Cyanidin-3-*O*-galactoside was the most unstable of the three investigated anthocyanins, its content being the most affected by the intestinal digestion compared to the crude extract. 

The pancreatic-bile digestion significantly decreased the antioxidant capacity of the anthocyanin-rich extract of Cornelian cherries. The ABTS•^+^ scavenging activity decreased by ~43%, as compared to the undigested or gastric digested sample. The changes in the antioxidant capacity of digested Cornelian cherry fruit anthocyanins were consistent with those previously observed for red cabbage anthocyanins [33]. While the bioaccessibility of Cornelian cherry anthocyanins largely decreased during intestinal digestion, the antioxidant capacity of the extract was less affected by this phase of digestion. This fact could be explained by the formation of new antioxidant metabolites from anthocyanins degraded from the simulated intestinal fluid, which can further exert their beneficial effects on human health. 

## 4. Conclusions

The present study evaluated for the first time the stability of anthocyanins from Cornelian cherries during their passage through the upper gastrointestinal tract by an in vitro simulation of the digestion process. The effect of the in vitro gastrointestinal digestion on the antioxidant capacity of the anthocyanin-rich extract was also investigated. The results indicated the presence of three anthocyanins, cyanidin-3-*O*-galactoside, pelargonidin-3-*O*-glucoside, and pelargonidin-3-*O*-rutinoside, in Cornelian cherry fruits, compounds that were not significantly affected by the gastric digestion. Intestinal digestion resulted in a large decrease of the anthocyanin content and antioxidant activity of the investigated fruit extract. These findings suggest that anthocyanins’ stability during gastrointestinal digestion should be taken into account when estimating their bioavailability. The consumption of Cornelian cherries may be an important source of anthocyanins in the human diet, which may exert their health beneficial effects at the gastric level, while their degradation products and metabolites may act as antioxidants in small intestine.

## Figures and Tables

**Table 1:** Anthocyanin identification and quantification (mg/L) of Cornelian cherry fruit extract samples before and after in vitro digestion.

Peak Number	Compound	Crude Extract	In Vitro Gastric Digestion	In Vitro Intestinal Digestion
1	Cyanidin-3-*O*-galactoside	128.45 ± 5.14 ^a^	137.52 ± 6.05 ^a^	29.9 ± 1.03 ^b^
2	Pelargonidin-3-*O*-glucoside	226.78 ± 8.61 ^a^	246.35 ± 10.12 ^a^	70.79 ± 3.13 ^b^
3	Pelargonidin-3-*O*-rutinoside	4.75 ± 0.15 ^a^	2.12 ± 0.09 ^b^	1.45 ± 0.06 ^b^
	Recovery (%)	100	107.23	26.46

^a,b^ Numbers in rows followed by different letters are significantly different (*p* < 0.05).
